# Dye Degradation, Antimicrobial Activity, and Molecular Docking Analysis of Samarium‐Grafted Carbon Nitride Doped‐Bismuth Oxobromide Quantum Dots

**DOI:** 10.1002/gch2.202300118

**Published:** 2023-11-10

**Authors:** Shams Rani, Muhammad Imran, Ali Haider, Anum Shahzadi, Anwar Ul‐Hamid, H. H. Somaily, Sawaira Moeen, Mahreen Khan, Walid Nabgan, Muhammad Ikram

**Affiliations:** ^1^ Department of Chemistry Government College University, Faisalabad Pakpattan Road Sahiwal 57000 Pakistan; ^2^ Department of Clinical Sciences Faculty of Veterinary and Animal Sciences Muhammad Nawaz Shareef University of Agriculture Multan 66000 Pakistan; ^3^ Department of Pharmacy COMSATS University Islamabad 54000 Pakistan; ^4^ Core research facilities King Fahd University of Petroleum & Minerals Dhahran 31261 Saudi Arabia; ^5^ Department of Physics Faculty of Science King Khalid University Abha 9004 Saudi Arabia; ^6^ Solar Cell Applications Research Lab Department of Physics Government College University Lahore Lahore 54000 Pakistan; ^7^ Departament d'Enginyeria Química Universitat Rovira i Virgili Av Països Catalans 26 Tarragona 43007 Spain

**Keywords:** catalytic, ciprofloxacin, co‐precipitation, doping, *Escherichia coli*

## Abstract

Various concentrations of samarium‐grafted‐carbon nitride (Sm‐g‐C_3_N_4_) doped‐bismuth oxobromide (BiOBr) quantum dots (QDs) are prepared by the co‐precipitation method. Elemental evaluation, morphological, optical, and functional group assessment are studied employing characterization techniques. Based on the XRD pattern analysis, it is determined that BiOBr exhibits a tetragonal crystal structure. The electronic spectroscopy revealed an absorption peak for BiOBr at 315 nm and the bandgap energy (*E*_g_) decreasing from 3.9 to 3.8 eV with the insertion of Sm‐g‐C_3_N_4_. The presence of vibrational modes related to BiOBr at 550 cm^−1^ is confirmed through FTIR spectra. TEM revealed that pure BiOBr possessed non‐uniform QDS, and agglomeration increased with the addition of Sm‐g‐C_3_N_4_. The catalytic performance of Sm‐g‐C_3_N_4_ into BiOBr (6 mL) in a neutral medium toward rhodamine B exhibited excellent results (99.66%). The bactericidal activity is evaluated against multi‐drug resistance (MDR) *Escherichia coli* once the surface area is increased by dopant and the measured inhibition zone is assessed to be 3.65 mm. Molecular docking results supported the in vitro bactericidal potential of Sm‐g‐C_3_N_4_ and Sm‐g‐C_3_N_4_ doped‐BiOBr as DNA gyrase*_E. coli_* inhibitors. This study shows that the novel Sm‐g‐C_3_N_4_ doped‐BiOBr is a better catalyst that increases specific semiconductor's catalytic activity (CA).

## Graphical abstract

Industrial effluent usually contains a combination of microorganisms, antibiotics, and dye contaminants. RhB catalytic degradation and inhibition of E. coli growth are achieved using synthesized Sm‐g‐C_3_N_4_ doped‐BiOBr QDs.

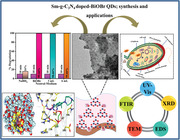

## 1 Introduction

Fresh water is crucial for all biological entities, but global contamination of current water resources has increased because of rapid industrialization and massive population growth.^[^ ^1^ ^]^ The presence of several dyes used in factories and present in contaminated water, such as methylene orange (MO), phenothiazine, triphenylmethane dyes, RhB, and methylene blue (MB), presents a significant risk to human health, disrupts the ecosystem and organisms attributed to their potential for being poisonous and carcinogenic.^[^ ^2^, ^3^ ^]^ Moreover, RhB (C_28_H_31_N_2_O_3_Cl) is a cationic dye highly soluble in water, belongs to the class of xanthenes, and irritates the skin, airways, and eyes.^[^ ^4^ ^]^ Inflammation of the lactating gland due to bacterial invasion is known as mastitis.^[^ ^5^ ^]^ The lactating gland has originated several microbial isolations.^[^ ^6^ ^]^ Conversely, *Staphylococcus aureus, Streptococcus agalactiae, Streptococcus uberis, Escherichia coli*, and additional *Streptococcus spp*. are often implicated as etiological entities in bovine mastitis.^[^ ^7^, ^8^, ^9^ ^]^ Antibacterial treatment of diseased animals relies on identifying the causative entities and their resistance tendencies, which is made more difficult by the global rise in resistance.^[^ ^10^ ^]^ Novel l pathways of antibiotic resistance are constantly occurring and proliferating worldwide, making AMR an increasingly severe problem. Certain illnesses are getting difficult to cure, if not impossible, as medications lose efficacy. As a result, the threat that antimicrobial resistance (AMR) presents to public health is escalating. Because of the potential for sickness, dwelling or performing near dairy animals.^[^ ^11^ ^]^ Classifies the ingestion of raw milk as a food‐borne hazard. Several nanoparticles (NPs) and related substances have garnered interest as possible antimicrobial agents. Nanostructures made of metallic elements like silicon (Si), silver (Ag), magnesium oxide (MgO), copper oxide (CuO), silver oxide (Ag_2_O), titanium dioxide (TiO_2_), calcium oxide (CaO), and zinc oxide (ZnO) are well known for antibacterial properties.^[^ ^12^, ^13^ ^]^ Resistance to commonly used antibiotics in various pathogens has prompted a search for alternative bactericidal substances.^[^ ^14^ ^]^ In purifying contaminated water to remove ions, numerous advanced traditional techniques, adsorption, photocatalysis, catalysis, membrane filtration,^[^ ^15^ ^]^ coagulation/flocculation, and advanced oxidation processes (AOP) were mainly employed;^[^ ^16^ ^]^ only a few have been accepted by the paper and textile fields.^[^ ^17^ ^]^ Catalysis is the most essential process based on nanomaterial semiconductors because of their low toxicity, chemical stability, and eco‐friendliness.^[^ ^18^ ^]^ NCs are now widely accessible and have a practical application for recycling contaminated water.^[^ ^19^ ^]^

Non‐TiO_2_ semiconductors such as bismuth oxyhalides (BiOX, X =  Br, I, and Cl) have gained widespread interest in wastewater remediation.^[^ ^20^ ^]^ BiOBr has recently triggered dye degradation due to its low Eg, environmental friendliness, and excellent chemical stability.^[^ ^20^, ^21^, ^22^ ^]^ However, using undoped BiOBr as a catalyst is still constrained by some limitations, including the relatively simple recombination of photogenerated electron‐hole (e^−^/h^+^) pairs and the limited utilization of the visible light spectrum. Furthermore, BiOBr NPs agglomerate, reducing active sites and less interaction between the catalyst and the pollutants.^[^ ^23^ ^]^ Xia et al.^[^ ^24^ ^]^ synthesized an MWCNT/BiOBr composite, reducing the RhB in 75 min. Jin et al. prepared BiOBr/Bi2S3/CdS exhibited 83.3% degradation against MeB within 120 min.^[^ ^25^ ^]^ Vadivel et al. synthesized Sm‐BiOBr/RGO and degraded the 94% MO after 70 min of visible light exposure.^[^ ^26^ ^]^ As a result, significant progress has recently been achieved with a couple of rare earth grafted carbon base materials to improve the dye elimination performance of Pure BiOBr at low time intervals. Incorporating Sm^3+^ can potentially enhance the solar energy conversion rate, while the unoccupied 5d and partially filled 4f orbitals can potentially improve the charge carrier separation rate.^[^ ^27^ ^]^ Carbon nitride is a metal‐free and visible‐light‐driven (470 nm) polymer that has piqued the interest of researchers due to its remarkable characteristics, such as earth abundance, physicochemical stability, strong biocompatibility, excellent physicochemical stability, and, most importantly, ease of preparation.^[^ ^28^, ^29^, ^30^ ^]^ Because of these properties, it has many applications in various fields, including water splitting,^[^ ^31^, ^32^ ^]^ organic contaminants removal,^[^ ^33^ ^]^ CO_2_ photo reduction,^[^ ^34^, ^35^ ^]^ and catalytic organic preparation.^[^ ^36^, ^37^ ^]^ It has been reported that the BiOBr‐g‐C_3_N_4_ heterojunctions effectively suppress the recombination of the e^−^/h^+^ pair, improving the dye degradation from wastewater.^[^ ^38^, ^39^, ^40^, ^41^, ^42^ ^]^ In recent decades, molecular docking predictions have been more popular as a key to understanding various biological processes. The significance of the biosynthetic pathway for nucleic acids in the discovery of antibiotics has been extensively documented.^[^ ^43^ ^]^ In recent years, a number of nanostructures have been demonstrated to be antibacterial,^[^ ^44^, ^45^ ^]^ but their precise mechanisms of action have yet to be determined. Sm‐g‐C_3_N_4_ and Sm‐g‐C_3_N_4_ doped‐BiOBr were attached, and their binding interactions and tendencies were compared to those of ciprofloxacin, the standard antibiotic used for evaluating antibacterial activity. The characteristic modification in BiOBr lattice with doping rare earth Sm‐grafted‐C_3_N_4_ has been expected as an excellent approach to gain batter CA at low time intervals against RhB, which to the extent feasible knowledge has not yet been published.

Based on the factors mentioned above, this study presents the first report on the economic co‐precipitation approach used to synthesize Sm‐g‐C_3_N_4_ doped‐ BiOBr QDs. The study used prepared samples to evaluate qualitative and quantitative RhB de‐colorization and bactericidal efficiency via molecular docking. Moreover, prepared samples were characterized with a variety of characterization techniques for the confirmation of QDs. A plausible justification for the formation of the QDs structure was discussed. The study also examined the influence of Sm‐g‐C_3_N_4_ content in Sm‐g‐C_3_N_4_ doped‐ BiOBr, solution pH, and catalyst quantity on CA. Furthermore, the reusability of as‐prepared nanocatalysts (NCs) was studied for up to five cycles via experiment. A potential mechanism of CA to eliminate the RhB is presented. In summary, the results of this paper should aid in developing improved catalysts for wastewater treatment.

## 2 Experimental Section

### 2.1 Materials

Sodium hydroxide (NaOH, 98%) and bismuth nitrate (Bi (NO_3_)_2_.5H_2_O, 98%) were acquired from Sigma Aldrich (Germany). Potassium bromide (KBr, 98%) and samarium nitrate (Sm (NO_3_)_3_.6H_2_O, 99.9%) were procured from UNICHEM and Alfa‐Aesar, respectively. Sodium borohydride (NaBH_4_), rhodamine B (RhB), and deionized water (DIW) were used. Carbon nitride (C_3_N_4_) was prepared in the lab through the pyrolysis of urea (CH_4_N_2_O). All the utensils were decontaminated using ethanol and entire chemicals were used in their original form.

### 2.2 Synthesis of Samarium‐g‐C_3_N_4_

**Figure 1:**
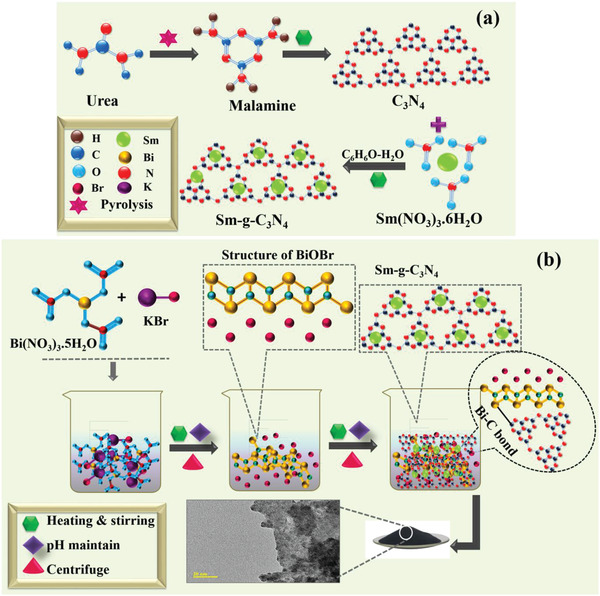
a) Schematic of Sm‐g‐C_3_N_4_ preparation. b) Sm‐g‐C_3_N_4_ doped‐BiOBr QDs.

The graphitic carbon nitride (g‐C_3_N_4_) was synthesized via pyrolysis of urea.^[^ ^46^ ^]^ An adequate amount of urea was immediately placed in a furnace at 550 °C for 5 h. This temperature transformed urea to melamine, producing a white powder of g‐C_3_N_4_. For grafting Sm to g‐C_3_N_4_, 60 mg of C_3_N_4_, and Sm(NO_3_)_3_.6H_2_O were dissolved in 60 mL of DI water under stirring for 4 h,7 mL of methanol was added as a sacrificial agent, as illustrated in **Figure** **1**a.

### 2.3 Synthesis of Sm‐g‐C_3_N_4_ Doped‐BiOBr

The co‐precipitation approach was employed to synthesize BiOBr QDs. Initially, 0.5 M of KBr (solution A) and 0.5 M of Bi (NO_3_)_3._5H_2_O (solution B) were synthesized separately at 60 °C for 20 min under robust stirring. Subsequently, added solution A drop by drop in solution B and observed the colloidal solution. After 1 h, 1 M of NaOH as a precipitating agent was introduced drop‐wise in the above colloidal solution to retain pH ≈12. To remove impurities such as nitrates, potassium, and sodium impurities, supernatants were wasted, and the sediments were acquired from the colloidal solution through centrifugation at 7000 rpm for 7 min repeatedly by DI water. Centrifuged sediments were heated at 150 °C for 12 h. The achieved BiOBr was grounded into fine powder. 3 and 6 mL Sm‐g‐C_3_N_4_ doped into BiOBr was synthesized using a similar procedure, Figure 1b.

### 2.4 Catalytic Activity (CA)

The degradation potency of RhB when exposed to NaBH_4_, Sm‐g‐C_3_N_4_ doped‐BiOBr QDs was investigated through CA. Firstly, a 0.01 M NaBH_4_ stock solution was prepared and ≈400 µL of NaBH_4_ solution was combined with 3 mL of RhB aqueous solution using a quartz cell. Moreover, the quartz cell was then filled with pristine and doped QDs. At constant time intervals, the de‐colorization of RhB was measured by electronic spectroscopy in 200–800 nm. The removal capacity was determined as follows.^[^ ^19^, ^47^ ^]^

(1) $$\%\;\mathrm{Degradation}=\frac{C_{o}-C_{t}}{C_{o}}\times 100$$

where *C*_o_ denotes the preliminary absorbance and *C*_t_ refers to the concentration of RhB at a specific period.

### 2.5 Isolation and Identification of MDR E. *coli*

#### 2.5.1 Sample Collection

Bovine milk specimens from clinically positive were obtained via direct milking in aseptic vitreous from chosen milch cattle marketed at several marketplaces and veterinary amenities throughout Pakistan. After collection at 4 °C, bovine milk was promptly carried to the lab. Coliforms identified in raw milk were enumerated on MacConkey agar, and all plates at 37 °C were incubated for 48 h.

#### 2.5.2 Identification and Characterization of Bacterial Isolates

Based on colonial morphology, *E. coli* had been identified through Gram staining and many biochemical assays following Bergey's Deterministic Bacteriology Manual.^[^ ^48^ ^]^

#### 2.5.3 Antibiotic Susceptibility

Bauer et al.^[^ ^49^ ^]^ used the disc diffusion approach to conduct an antibiotic susceptibility test on Mueller Hinton Agar (MHA). The experiment was designed to investigate *E. coli* antibiotic resistance: Imipenem (Imi) 10 µg (carbapenem), ciprofloxacin (Cip) 5 µg (quinolones), amoxicillin (A) 30 µg (penicillins), ceftriaxone (Cro) 30 µg (cephalosporins), tetracycline (Te) 30 µg (tetracyclines), azithromycin (Azm) 15 µg (macrolides), and gentamicin (Gm) 10 µg (aminoglycosides).^[^ ^50^ ^]^ Cleaned *E. coli* culture was grown and adapted to 0.5 MacFarland opacity. On Muller Hinton Agar (MHA), the antibiotic discs were positioned distant from the top of the incubation dish to protect the inhibitory zones from overlapping (Oxoid Limited, Basingstoke, UK). Inoculation of plates for 24 h at 37 °C and the outcomes were evaluated corresponding to the Clinical & Laboratory Standard Institute.^[^ ^51^ ^]^ A bacterium resistant to at least three antibiotics was identified as MDR.^[^ ^52^ ^]^

#### 2.5.4 Antimicrobial Activity

The antimicrobial potency of BiOBr and Sm‐g‐C_3_N_4_ doped‐BiOBr QDs against MDR *E. coli* isolates acquired from bovine or caprine mastitic milk employing agar well diffusion technique. On (MacConkey agar) MA, Petri plates were swabbed with 1.5 108 CFU/ mL^−1^ (0.5 McFarland standard) MDR *E. coli*. Wells with 6 mm diameters were formed by sterilized cork borer. Many doses of as‐prepared samples were used as (1.0 mg/50 L) and (0.5 mg/50 L). DIW (50 L) and ciprofloxacin (0.005 mg/50 L) functioned as the negative and positive control, respectively.^[^ ^53^ ^]^

#### 2.5.5 Statistical Analysis

Statistical analysis of inhibitory zone sizes was conducted using SPSS 23 by one‐way analysis of variance (ANOVA), and bactericidal effectiveness was evaluated based on inhibition zone size (mm).^[^ ^54^ ^]^

### 2.6 Molecular Docking Analysis

Sm‐g‐C_3_N_4_ and Sm‐g‐C_3_N_4_‐doped BiOBr nanocomposites were evaluated for their binding capacity to specific enzyme targets. Regarding binding capabilities, Sm‐g‐C_3_N_4_ and Sm‐g‐C_3_N_4_ doped BiOBr NSs were compared to ciprofloxacin (standard antibiotic inhibits bacterial DNA gyrase). With the increased bactericidal activity of generated NPs against *E. coli*, the binding potential inside the active region of DNA gyrase was examined. The DNA gyrase*_E.coli_* target enzyme structures were downloaded from the protein data bank using accession number 5MMN (Res: 1.9).^[^ ^55^ ^]^

Molecular docking predictions were performed using Sybyl X‐2.0^[^ ^44^, ^56^ ^]^ using sketch module‐created ligand structures. To accomplish energy conservation, water molecules with their native ligands were eliminated, polar H‐atoms were later added to each molecule, and the system was neutralized. It was established that the distance between the endogenous ligand and the binding pocket is less than 5. The top 10 docked complexes were chosen for further analysis in each instance. Using Pymol, a 3D model of the binding interactions between molecules was created.

### 2.7 Characterization of Synthesized Doped BiOBr

The crystalline character and phase structure of Sm‐g‐C_3_N_4_‐doped BiOBr were verified employing a PAN analytical X'pert PRO powder diffractometer equipped with monochromatic Cu‐Kα radiations (*λ* ∼ 0.0154 nm) in 2θ range of 10°–80°. The optical properties of prepared pure and doped BiOBr were probed using electronic spectroscopy LABDeX, which covered a range of 200–600 nm. High‐resolution transmission electron microscopy (HR‐TEM, JEM2100F, JEOL, Japan) and FESEM (JSM‐6460LV) paired with an EDX spectrometry were employed to assess the lattice fringes, elemental constituents, and morphology of Sm‐g‐C_3_N_4_‐doped BiOBr. FTIR spectrophotometer and a PerkinElmer 3100 were adapted to analyze the functional groups and their variation of prepared specimens.

## 3 Results and Discussion

As demonstrated in Figure 1, the co‐precipitation method was adopted to dope the various concentrations (3 and 6 mL) of synthesized Sm‐g‐C_3_N_4_ in BiOBr QDs.

**Figure 2:**
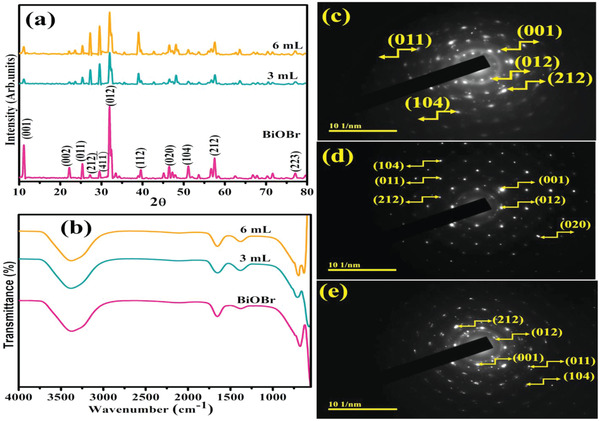
a) XRD pattern b) FTIR spectra, c–e) SAED images of pristine BiOBr, and (3 and 6 mL) Sm‐g‐C_3_N_4_ doped‐QDs.

Phase identification, crystallographic planes, and crystal structure of synthesized QDs were obtained in XRD patterns in the 2θ (10–80°) range as expressed in **Figure** **2**a. Diffraction peaks for the tetragonal structure of BiOBr located at 10.8, 21.9, 25.3, 31.8, 39.4, 46.3, 50.6, 57.3, and 77.4° analogs to (001), (002), (011), (012), (112), (020), (104), (212), and (223) reflection planes respectively, authenticated by (JCPDS Card No. 01‐073‐2016, 01‐085‐0862). Flexing of peaks at 27.0° (212) and 29.6° (41$\bar{1}$) corroborated the monoclinic geometry of Bi_4_Br_2_O_5_ synchronized with (JCPDS Card No. 00‐037‐0699). Figure S1, Supporting Information depicts a Rietveld refinement profile containing XRD data of as‐prepared QDs. The dots represent the experimental data, whereas the solid line indicates the Rietveld refinement fit. The bottom line (red color) represents the difference between the observed and estimated values at each stage. The cell parameters are *a* = 3.92 Å, *b* = 3.92 Å, *c* = 8.11 Å, and the volume of the cell is 124.62 A^3^. No additional peaks of dopants (Sm and C_3_N_4_) were identified in Sm‐g‐C_3_N_4_ doped‐BiOBr, suggesting that the minimal dopant concentration and may be dopant uniformly dispersed in the BiOBr surface which does not modify the phase of BiOBr QDs. Diffraction peak intensity is reduced after Sm‐g‐C_3_N_4_ doping, indicating that Sm slows the growth rate and confines the crystallization.^[^ ^57^ ^]^ The average crystallite size of the as‐synthesized specimens has been estimated utilizing XRD data with the application of the Scherer equation.^[^ ^58^ ^]^

(2) $$D=\frac{K\lambda }{\beta \cos\theta }$$

where the crystallite size (*D*) is determined in nanometers (nm). The above equation takes into account the wavelength (*λ*) of the incident radiation (measured in nm), a constant value (*k*) usually set to 0.89, the diffraction angle (θ), and the peak width at half maximum (*β*). The average crystallite size for bare BiOBr is 27 nm, reduced upon doping from 27 to 24 nm. Moreover, the Williamson–Hall (W–H) model computed the crystallite size, which is 24.6 nm for pure BiOBr, and decreased with the insertion of a dopant from 24.6 to 22.7 nm. The data revealed that the crystallite size decreased irrespective of the method used. However, it is noteworthy that the W–H method consistently provides smaller crystallite sizes compared to the values obtained from the Scherer method. The decrement in the crystallite size of Sm‐g‐C_3_N_4_ doped‐BiOBr was attributed to the variation in the dopant and host material ionic radii.^[^ ^59^ ^]^

FT‐IR spectra revealed the elemental compositions and functional group evaluation of undoped and Sm‐g‐C_3_N_4_ doped‐BiOBr. Various bands were observed in the 4000–400 cm^−1^ region as elaborated in Figure 2b. The H–O–H bending vibration mode, which can be attributable to the moisture, was assigned to the band centered at 1650 cm^−1^.^[^ ^60^ ^]^ Transmittance bands at 3460 and 1380 cm^−1^ can be associated with the O–H bond and carboxyl O–H stretching vibration, respectively.^[^ ^61^, ^62^ ^]^ The peak flexing at 550 cm^−1^ exposed the stretching vibration of Bi–O, confirming the synthesis of BiOBr QDs.^[^ ^60^ ^]^ The stretching vibration of the Bi–O bond is related to BiOBr spectra in the 600–1000 cm^−1^ region.^[^ ^63^ ^]^ It was perceived that a notable shift towards lower wavenumber occurred with the inclusion of dopant. This shifting raised the bond length as the variation in bond length is linked to a shift in the electronegativity of the surrounding atom. It has been observed in the literature that changes in the size, shape, and local defects of NPs can lead to modifications in both the position and width of FTIR peaks.^[^ ^64^ ^]^

The SAED analysis disclosed the diffraction rings related to the (012), (001), (212), (011), (104), and (020) reflection planes of BiOBr and doped BiOBr well match with XRD results presenting the poly‐ crystallinity of QDs (Figure 2c–e).

**Figure 3:**
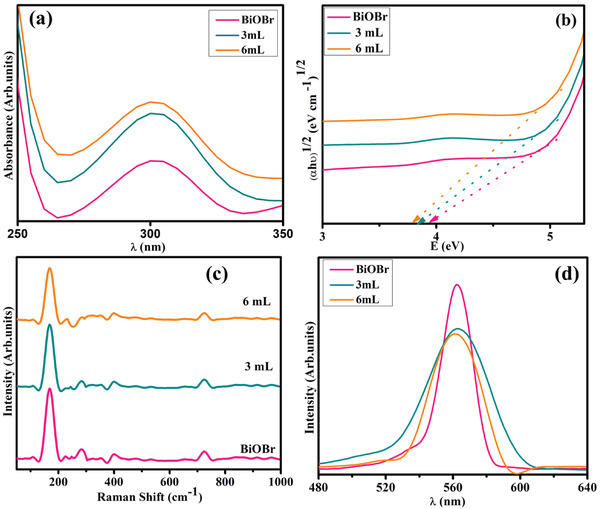
a) Electronic spectra of samples b) *E*_g_ plot of synthesized doped BiOBr, c) Raman spectra, and d) emission spectra of pure and (3 and 6 mL) Sm‐g‐ C_3_N_4_ doped‐BiOBr.

Electronic spectroscopy was utilized to investigate the optical characteristics of pristine and Sm‐g‐C_3_N_4_ doped‐BiOBr QDs. The absorption of BiOBr has suggested the absorption peak around 315 nm might be ascribed to the π–π* transition^[^ ^33^, ^34^ ^]^ as represented in **Figure** **3**a. The absorption increased gradually with increasing amount of Sm‐g‐C_3_N_4_ to BiOBr. Tauc's plot has been employed to evaluate the E_g_ of synthesized specimens (Figure 3b). According to the peak mentioned above, the optical E_g_ of pristine BiOBr was determined to be 3.94 eV and reduced for (3 and 6 mL) Sm‐g‐C_3_N_4_ doped QDs. Band gap reduction reveals the stoichiometry deviation and degeneracy of doped BiOBr as well as an increase in oxygen vacancies inside the lattice.^[^ ^35^ ^]^ Additionally, the incorporation of C_3_N_4_ caused the lowering of the E_g_ attributed to the chemical alteration of C_3_N_4;_ it may have increased charge carriers’ capacity to absorb and transition.^[^ ^65^ ^]^

Raman spectroscopy is a vibrant tool due to its adequate spatial resolution to assess the microscopic structure and flaws of BiOBr and Sm‐g‐C_3_N_4_ doped‐BiOBr species. Raman spectra have been depicted in Figure 3b, consisting of characteristic bands at 169, 398, and 717 cm^−1^. The band at ≈167 cm^−1^ may be assigned to the internal stretching of BiOBr bonds, which is associated with the E_g_ mode of BiOBr.^[^ ^66^ ^]^ The E_g_ and B_1g_ modes produced through the movement of O atoms have been designated to the band at 393 cm^−1^.^[^ ^66^ ^]^ The band at 717 cm^−1^ was ascertained in the published research.^[^ ^67^ ^]^ A blue shift has been noticed in Raman peaks upon incorporating Sm and C_3_N_4_. This behavior is prevalent in NPs and may be described using phonon and quantum confinement concepts.^[^ ^68^, ^69^ ^]^ Moreover, the intensity of the peaks reduced after the inclusion of a high concentration of Sm and C_3_N_4_ (6 mL Sm‐g‐ C_3_N_4_ doped‐BiOBr), which can be ascribed to the distortion in the lattice of BiOBr.^[^ ^70^ ^]^

Charge‐carriers separation efficacy of BiOBr and (3 and 6 mL) Sm‐g‐ C_3_N_4_ doped‐BiOBr QDs were exploded via PL analysis (Figure 3d). BiOBr emission spectrum depicted at 560 nm, signifying a considerable probability of e^−^/h^+^ recombination.^[^ ^71^ ^]^ Upon incorporating the dopant Sm‐g‐C_3_N_4_ doped‐BiOBr into BiOBr, the PL intensities exhibit an emission edge resembling that of the host BiOBr but notably reduced. This reduction in intensities can be attributed to the interaction between C_3_N_4_ and BiOBr, which may help to minimize the e^−^/h^+^ recombination and accelerate the charge separation.^[^ ^72^ ^]^ The shallow traps developed by adding further lattice defects and defect levels upon doping of Sm weakened the emission intensities, which is one of the leading causes that inhibit prepared QDs exciton recombination rate.^[^ ^73^ ^]^ The prepared NCs Sm‐g‐C_3_N_4_ doped‐BiOBr QDs are good candidates to de‐colorize the RhB.

EDS mapping investigates the chemical composition to validate the purity of the synthesized catalysts illustrated in Figure S2a–c, Supporting Information. The strong peak of Bi, O, and Br observed in spectra indicates the existence of BiOBr. The usage of NaOH to sustain the pH accountable for Na peaks. The Au peak suggests that the sample had a coating sprayed on it, providing consistent conductivity and a uniform surface for examination. It avoids the charged surface and assists the e^−^s field emission. Al peak identified, used to coat the specimens. Contamination can be assigned by generating *K* peak signals. Ytterbium (Yb) emerged due to operator error.

**Figure 4:**
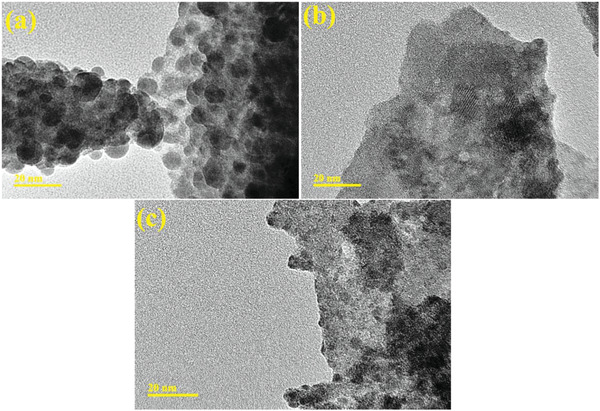
TEM images of a) BiOBr, and b,c) Sm‐g‐C_3_N_4_ doped‐BiOBr at 3 and 6 mL, respectively.

TEM and HR‐TEM analyzed morphology and the topography of bare and doped BiOBr. The control sample BiOBr has non‐uniform QDs morphology and showed agglomeration as the water was the solvent. The calculated average particle size was 9.25 nm, as represented in **Figure** **4**a. Upon doping of Sm‐g‐C_3_N_4_ (3 mL) into BiOBr (Figure 4b), a high level of aggregation was observed, and it looks like QDs overlapped by the dopants sheets indicating the significant interfacial interaction between dopant and BiOBr. Figure 4c shows the higher dopant concentration (6 mL) in BiOBr, confirming the over‐layered dopants seem that QDs are merged.

HR‐TEM images calculated interlayer d‐spacing with 10 nm resolution. Lattice spacing was found to be 0.40, 0.35, and 0.32 nm for control and (3 and 6 mL) Sm‐g‐C_3_N_4_ doped‐QDs, respectively, as disclosed in Figure S3a–c, Supporting Information, which are correlated with XRD. TEM unveiled that Sm‐g‐C_3_N_4_ is overlaid on QDs and compatible with HR‐TEM as lattice fringes were reduced upon doping of Sm‐g‐C_3_N_4_.

In the first degradation step, NaBH_4_ ionized into H^+^ (e^−^ acceptor) and BH_4_^−^ (e^−^ donor) ions in the redox reaction. Only in the presence of NaBH_4_ degradation response was incredibly slow. To enhance the rate as well as stability of the reaction, NCs introduced with NaBH_4_. This combination of having extensive surface atomic coordination influences the breakdown efficiency since it provides more active sites. Generally, dye and BH_4_^−^ were absorbed strongly on the immense surface of NCs due to hydrogen bonding, π–π bond, and molecular interaction.^[^ ^74^ ^]^ Cationic dye gains hydrogen (H) atoms and e^−^s from BH_4_; hence, double bonds break between aromatic rings and dye nitrogen (N) atoms. Attachment of e^−^s and H atoms with N^+^ to occupy the vacancy of a broken bond, resulting in π‐bond conjugation. NCs facilitate the shifting of e^−^s quickly from NaBH_4_ to dye; resultantly, RhB is converted into corresponding reduced form Leuco RhB (LRhB) and de‐colorized. Subsequently, BH_4_^−^ and LRhB were desorbed from the NCs’ surface. Dye breakdown is directly related to the concentration of NCs; an increase in the dopant concentration raises the degradation significantly, while at low concentrations, dyes exhibit modest degradation, Figure S4, Supporting Information. When NCs were incorporated, e^−^/h^+^ separation and transfer mechanisms  improved in terms of catalytic applications.^[^ ^75^ ^]^

**Figure 5:**
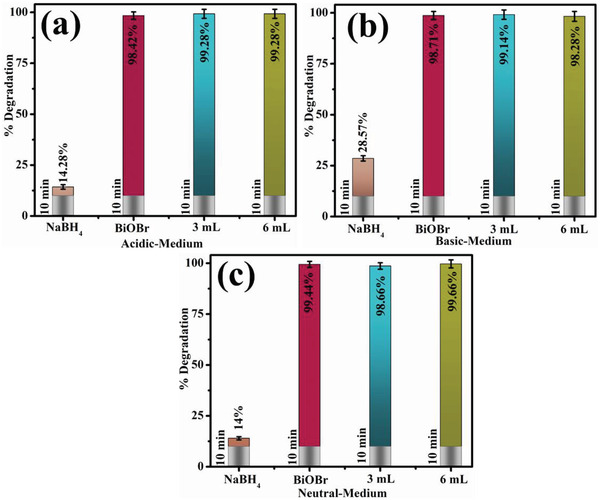
CA of pristine and (3 and 6 mL) Sm‐g‐C_3_N_4_ doped‐BiOBr in a) acidic, b) basic, and c) neutral media.

Electronic spectroscopy analyzed the CA of undoped and Sm‐g‐C_3_N_4_ doped‐BiOBr QDs in the presence of NaBH_4_ for RhB de‐colorization at different pH levels (acidic, basic, and neutral environments). The maximum degradation of control and Sm‐g‐C_3_N_4_ (3 and 6 mL) doped‐BiOBr QDs was 98.42%, 99.28%, and 99.28% in an acidic environment (pH = 4), 98.71%, 99.14%, and 98.28% in the basic environment (pH = 12), as well as 99.44%, 98.66%, and 99.66% in a neutral environment (pH = 7) as depicted in **Figure** **5**a–c. The crystallite size, shape, and surface area of the NCs significantly impact CA. H_2_O_2_ and O^2‐^ are inorganic oxidants present in synthesized samples. These oxidants boost the number of trapped e^−^s, preventing recombination and producing oxidizing radicals, which may improve catalytic potential. The dye pH solution is crucial to the entire adsorption process.^[^ ^76^ ^]^ The pH of the solution affects the sorption–desorption processes and the separation e^−^/h^+^ on the surface of the semiconductor. The degradation rate increased as the concentration of NCs or quantity of dye increased. The UV light wavelength and intensity influence the dye degradation in aqueous solution.^[^ ^77^ ^]^ The better degradation was noted in an acidic environment, ascribed to the raised formation of H^+^ ions available for desorption on the NCs’ surface.^[^ ^78^ ^]^ In a basic environment, CA demonstrated good results according to increased electrostatic interaction between the catalysts (negatively charged) and dye (positively charged).^[^ ^79^ ^]^ The inhibitory impact appears to be more significant at pH 7, possibly attributable to two primary forms of RhB in water zwitter ionic (RhB±) and cationic (RhB+). Consequently, electrostatic repulsion between the dye and the catalyst in both acidic and basic repulsion mediums led to lower CA of Sm‐g‐C_3_N_4_ doped‐BiOBr compared to a neutral environment.^[^ ^80^ ^]^ Adding dopants improved the degradation assigned to the presence of more active sites, providing a large surface area for the catalyst. The addition of Sm revealed the greatest degradation rate in all media because it boosted oxygen storage ability and catalytic efficiency.^[^ ^81^ ^]^ It has been reported that C_3_N_4_ displayed considerable degradation potency to RhB than other catalysts over a short time.^[^ ^82^ ^]^ Table S2, Supporting Information represents a comparison of the present research with the literature.

The regeneration of the catalyst is paramount to assess its efficacy for the effluent treatment. The recyclability of Sm‐g‐C_3_N_4_ doped‐BiOBr was evaluated via repetitive experiments as demonstrated in Figure S5, Supporting Information. Before each subsequent round, the catalysts were recovered from the de‐colorized dye solution, washed, dried, and then exposed to dye degradation for five turns. Even after five rounds, the efficiency of the reused catalyst was statistically significant, indicating the stability of the Sm‐g‐C_3_N_4_ doped‐BiOBr catalyst. A minor reduction in the de‐colorized rate of RhB can be attributed to the sequential washing and drying. Moreover, the stability of 6 mL Sm‐g‐C_3_N_4_ doped‐BiOBr has been assessed employing XRD before and after the catalytic reaction as illustrated in Figure S6, Supporting Information. XRD pattern of Catalyst after the reaction exhibits no notable modification regardless of a reduction in peak intensity. Sm‐g‐C_3_N_4_ doped‐BiOBr QDs crystal structure was not devastated.^[^ ^83^ ^]^

Employing a well diffusion assay for MDR *E. coli*, the bactericidal action of synthesized samples was assessed via inhibition ranges (mm). Estimating inhibition zone was measured from (2.05 ± 0.03– 3.65 ± 0.01 mm) to (1.55 ± 0.04– 3.15 ± 0.02 mm) in MDR *E. coli* at maximal and minimal doses accordingly against ciprofloxacin (positive control) and DIW (negative control), as depicted in Figure S8a,b, Supporting Information. **Table** **1** unveiled the significant (*p < 0.05*) antibacterial competence; Sm‐g‐C_3_N_4_ doped‐QDs showed the optimal microbial vulnerability compared to BiOBr. Active oxygen vacancies of Sm^3+^ might lead to the production of ROS and oxygen stress, which causes microbes to die, which is a plausible explanation for better antibacterial efficacy.^[^ ^84^ ^]^ In Sm‐g‐C_3_N_4_ doped‐BiOBr, C_3_N_4_ is the e^−^s receiver, and the charge carriers separate faster, lowering the recombination rate. The longer lifespan of e^−^/h^+^ pairs produces more radicals, which enhance the antibacterial action of Sm‐g‐C_3_N_4_ doped‐BiOBr.^[^ ^85^ ^]^ Table S3, Supporting Information elucidated a comparison of the antibacterial activity of the present research with the previous study.

**Table 1:** Microbicidal efficiency of undoped and (3 and 6 mL) Sm‐g‐C_3_N_4_ doped‐QDs.

Samples	Inhibition areas [mm]	
	0.5 mg/50 µL	1.0 mg/50 µL
BiOBr	1.55 ± 0.04	2.05 ± 0.03
3 mL	2.25 ± 0.03	2.95 ± 0.02
6 mL	3.15 ± 0.02	3.65 ± 0.01
Ciprofloxacin	6.40 ± 0.01	6.40 ± 0.01
DI water	0 ± 0.0	0 ± 0.0

As elaborated in Figure S9, Supporting Information, the formation of ROS (OH, HO_2_, O_2,_ and H_2_O_2_), the production of free radicals, and the viability of biological membranes have all been mainly associated with bactericidal efficacy. The capacity of semiconductors to provide e^−^s resulted in ROS formation. The bacterial cell wall has nm‐sized pores that allow NPs of the proper size and charge to pass through. These NPs insert cell membrane devastation by interacting with proteins and DNA, ultimately denaturing cell function. NPs increase ROS to assist in the formation of inhibitory zones.^[^ ^86^ ^]^

**Figure 6:**
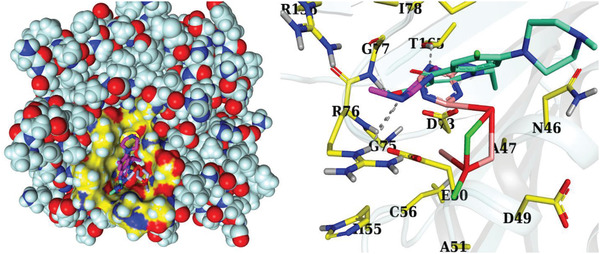
3D representation of ciprofloxacin, and native ligand binding inside binding pocket of DNA gyrase*_E. coli_*.

Molecular docking studies, in particular, have attracted much attention over the last several decades and have made it feasible to conduct in depth research into the mechanisms that underlie a wide range of biological activities. In the present study, molecular docking was used to evaluate the binding ability and inhibitory potential of synthesized NPs. This strategy aimed to suggest potential inhibitors that may be used against certain enzyme targets. DNA gyrase, a key enzyme in bacterial survival and development, has been identified as a promising drug target.^[^ ^87^ ^]^ Sm‐g‐C_3_N_4_ and Sm‐g‐C_3_N_4_ doped‐BiOBr were docked, and their binding interactions and tendencies were compared to those of ciprofloxacin, a standard antibiotic used in antibacterial activity testing (**Figure** **6**).

**Figure 7:**
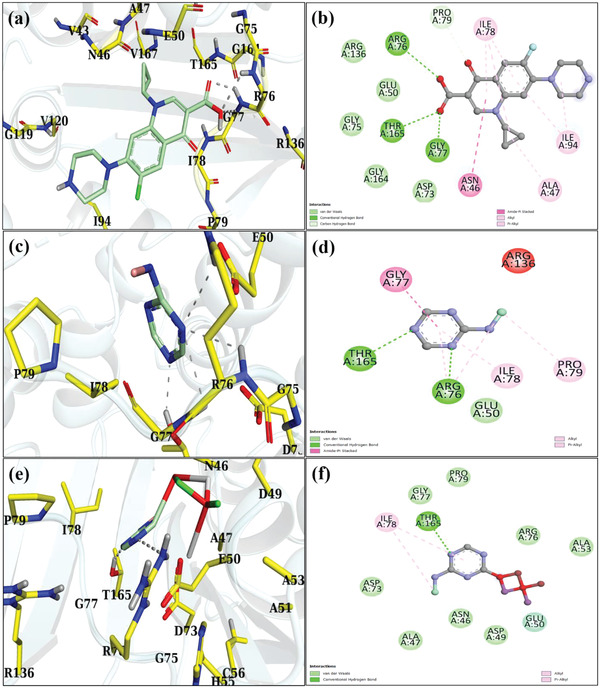
Binding interactions with a,b) ciprofloxacin, c,d) Sm‐g‐C_3_N_4_, and e,f) Sm‐g/C_3_N_4_‐doped BiOBr inside the active pocket of DNA gyrase*_E. coli_*.

For DNA gyrase*_E. Coli_*, binding scores of 2.43 and 3.51 were found for Sm‐g‐C_3_N_4_ and Sm‐g/C_3_N_4_‐doped BiOBr NPs, respectively. Sm‐g‐C_3_N_4_ doped‐BiOBr NPs had a binding score similar to that of the antibiotic ciprofloxacin with a binding score of 6.7 (**Figure** **7**a,b). The largest contribution to the docked complex formation of Sm‐g‐C_3_N_4_ NSs (Figure 7c,d) came from two H‐bonds, namely Arg76 and Thr165, and three hydrophobic interactions with Gly77, Ile78, and Pro79. While DNA gyrase*_E. Coli_* complexed with Sm‐g‐C_3_N_4_ doped‐BiOBr NPs displayed H‐bond with Thr165 and hydrophobic contacts with Ile 78, as depicted in Figure 7e,f.

## 4 Conclusion

Pristine and (3 and 6 mL) Sm‐g‐C_3_N_4_ doped‐BiOBr were efficiently synthesized through a co‐precipitation route to test antibacterial action and catalytic potency. The XRD spectra indicated that BiOBr had a tetragonal structure with crystalline nature. FTIR disclosed the transmittance peak at 550 cm^−1^ designating to the BiOBr, while bright dots determined crystallinity in SAED. Electronic spectroscopy depicts a significant decrease in E_g_ from 3.9 to 3.8 eV upon doping. Lattice *d*‐spacing was computed for control and doped BiOBr 0.40, 0.35, and 0.32 nm, respectively. The maximum degradation of 99.61% was demonstrated by adding BiOBr with (6 mL) Sm‐g‐C_3_N_4_ in a neutral medium against the RhB. Furthermore, the microbicidal potency of the QDs against clinically positive bovine mastitogen MDR G^−ve^ pathogen was investigated with an inhibitory zone ranging from 2.05 ± 0.03–3.65 ± 0.01 mm at maximal concentration. Molecular docking investigations revealed that Sm‐g‐C_3_N_4_ and Sm‐g‐C_3_N_4_ doped‐BiOBr NPs from *E. coli* might act as potential inhibitors of DNA gyrase enzymes. On top of that, this prepared specimen has a remarkable aptitude for cationic dye (RhB). This research contributed a mild technique to synthesize BiOBr‐based ternary composites for the enhancement of CA, thus pointing to the new strategy to improve RhB degradation further. Moreover, further advanced research in the coupling of BiOBr‐based NCs is expected, with the goal of developing novel composites with proper *E*_g_, long‐term stability, and surface features that might revolutionize the removal of pollutants from wastewater. This study will serve as a roadmap for future researchers, directing their endeavors in the area of developing efficient NPs specifically designed for water remediation.

## Conflict of Interest

The authors declare no conflict of interest.

## Supporting information

Supporting Information

## Data Availability

The data that support the findings of this study are available from the corresponding author upon reasonable request.
