# Correction to “Dye Degradation, Antimicrobial Activity, and Molecular Docking Analysis of Samarium‐Grafted Carbon Nitride Doped‐Bismuth Oxobromide Quantum Dots”

**DOI:** 10.1002/gch2.70087

**Published:** 2026-02-09

S. Rani, M. Imran, A. Haider, et al., “Dye Degradation, Antimicrobial Activity, and Molecular Docking Analysis of Samarium‐Grafted Carbon Nitride Doped‐Bismuth Oxobromide Quantum Dots.” *Global Challenges* **2023**, *7*, 2300118. https://doi.org/10.1002/gch2.202300118.

**The following text should be added at the start of section 2.3**:

BiOBr was synthesized and characterized following our previous study. Few characterizations reused in the present study.^[46a]^

We apologize for this error.

**Reference**

[46a.] Yousaf, Muhammad Shahid, Ali Haider, Anum Shahzadi, Anwar Ul‐Hamid, Muhammad Imran, Muhammad Ali Khan, Walid Nabgan, Murefah Mana Al‐Anazy, E. El Shiekh, and Muhammad Ikram. “Aggrandized Catalytic and Bactericidal Activity of Silver and Polyvinylpyrrolidone Capped Bismuth Oxybromide Quantum Dots: In Silico Molecular Docking Studies,” *Journal of Inorganic and Organometallic Polymers and Materials* 34, no. 2 (2024): 533‐545. DOI: https://doi.org/10.1007/s10904‐023‐02821‐7, https://doi.org/10.1002/gch2.202300118

