# Supplementary material for: Dye Degradation, Antimicrobial Activity, and Molecular Docking Analysis of Samarium‐Grafted Carbon Nitride Doped‐Bismuth Oxobromide Quantum Dots
Source: Glob Chall. 2023 Nov 10;7(12):2300118. doi: 10.1002/gch2.202300118 (PMC10714022; doi:10.1002/gch2.202300118)
Supplement: Supplementary file 1 — Supporting Information [file GCH2-7-2300118-s001.pdf]

Global Challenges
Open Access

Supporting Information

for *Global Challenges.*, DOI 10.1002/gch2.202300118

Dye Degradation, Antimicrobial Activity, and Molecular Docking Analysis of Samarium-Grafted Carbon Nitride Doped-Bismuth Oxobromide Quantum Dots

Shams Rani, Muhammad Imran, Ali Haider, Anum Shahzadi, Anwar Ul-Hamid, H. H. Somaily, Sawaira Moeen, Mahreen Khan, Walid Nabgan* and Muhammad Ikram**

Dye degradation, antimicrobial activity, and molecular docking analysis of samarium-grafted carbon nitride doped-bismuth oxobromide quantum dots

Shams Rani[a], Muhammad Imran[a*], Ali Haider[b], Anum Shahzadi[c], Anwar Ul-Hamid[e], H. H. Somaily[f], Sawaira Moeen[d], Mahreen Khan[d], Walid Nabgan[g*], Muhammad Ikram[d*]

[a]Department of Chemistry, Government College University, Faisalabad, Pakpattan Road, Sahiwal, Punjab, 57000, Pakistan
[b]Department of Clinical Sciences, Faculty of Veterinary and Animal Sciences, Muhammad Nawaz Shareef, University of Agriculture, Multan 66000, Punjab, Pakistan
[c]Department of Pharmacy, COMSATS University, Islamabad, Lahore Campus, 54000, Pakistan
[d]Solar Cell Applications Research Lab, Department of Physics, Government College University Lahore, Lahore 54000, Punjab, Pakistan
[e]Core research facilities, King Fahd University of Petroleum & Minerals, Dhahran 31261, Saudi Arabia
[f]Department of Physics, Faculty of Science, King Khalid University, P.O. Box 9004, Abha, Saudi Arabia
[g]Departament d'Enginyeria Química, Universitat Rovira i Virgili, Av Països Catalans 26, 43007 Tarragona, Spain

Corresponding author's email: [d]dr.muhammadikram@gcu.edu.pk, [a]imran@mail.ipc.ac.cn, [g]wnabgan@gmail.com

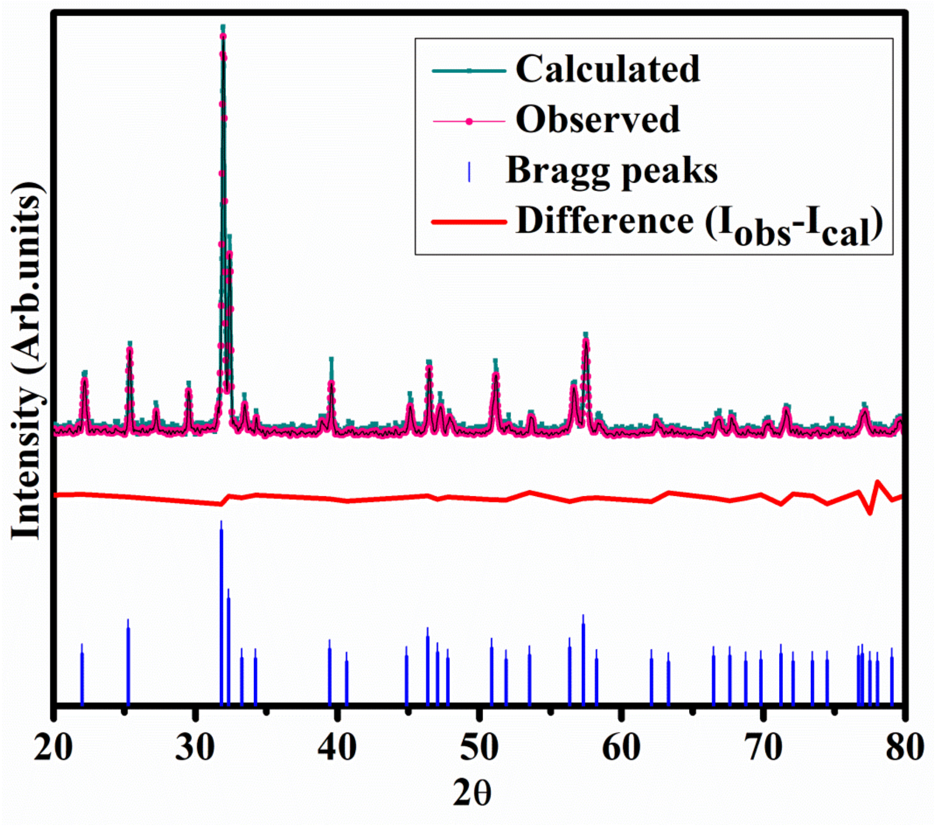

Fig. S1: Rietveld refinement profile of XRD data of prepared BiOBr QDs

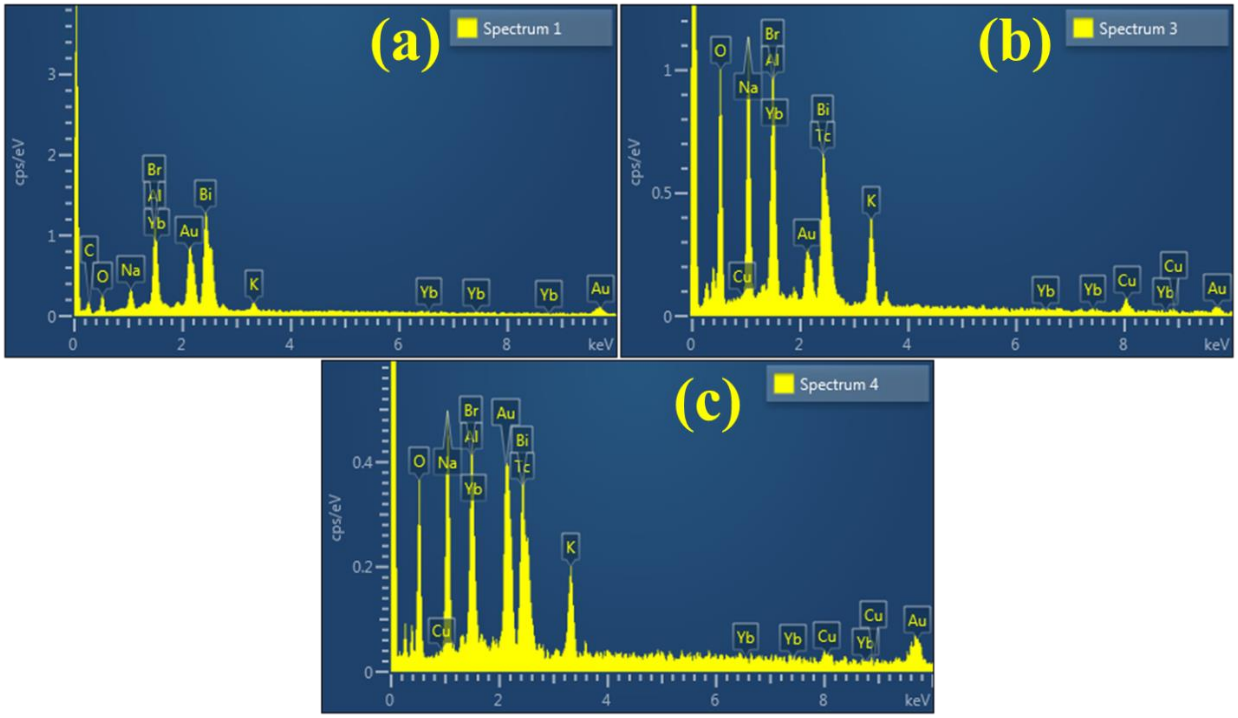

Fig.S2: EDS patterns of (a) bare BiOBr (b) 3 mL Sm-g-C_3N_4 doped-BiOBr QDs(c) 6 mL Sm-g-C_3N_4 doped-QD

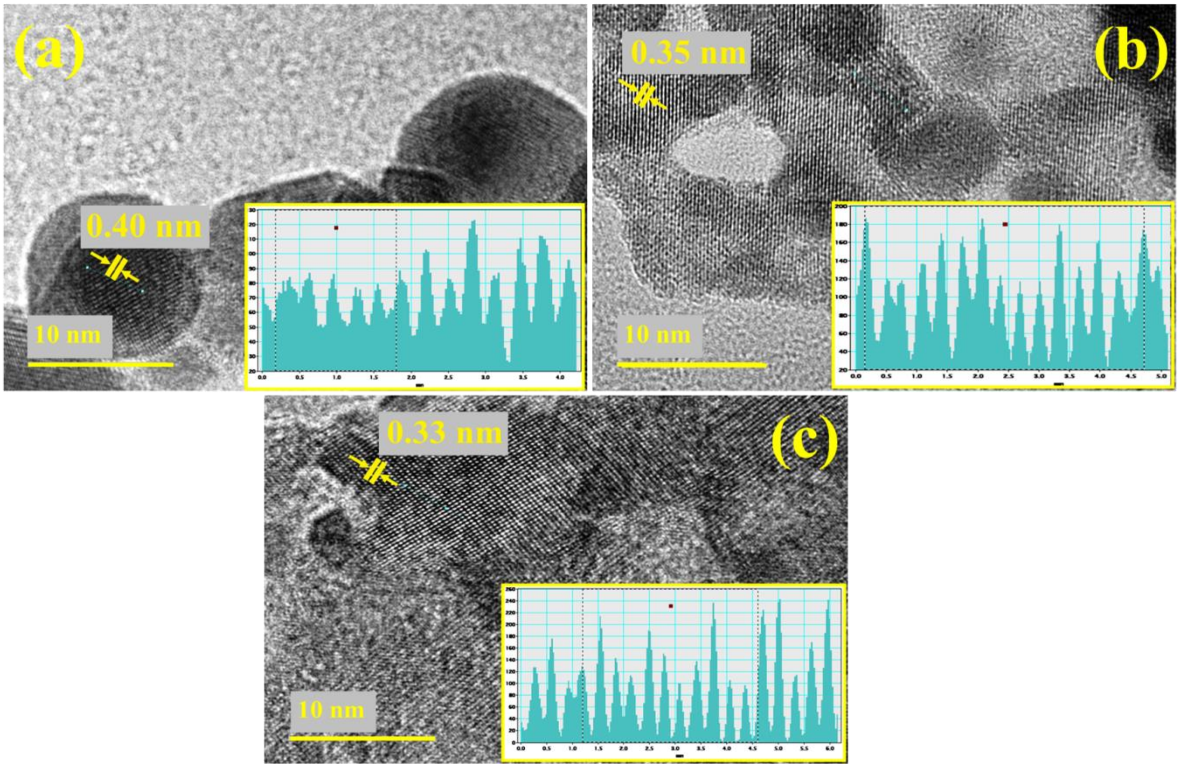

Fig.S3: (a-c) interlayer d-spacing of pristine and (3 and 6 mL) Sm-g-C_3N_4 doped-BiOBr

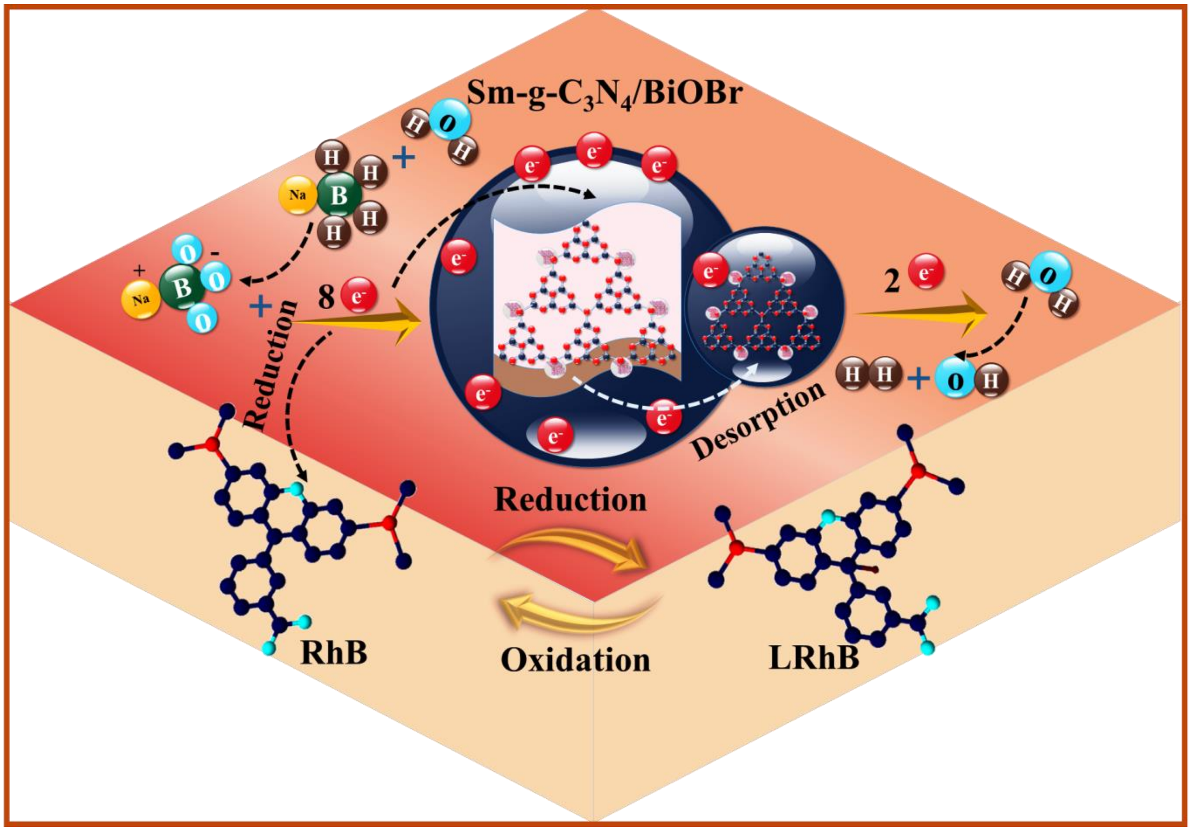

Fig.S4: Catalysis mechanism of prepared doped QDs

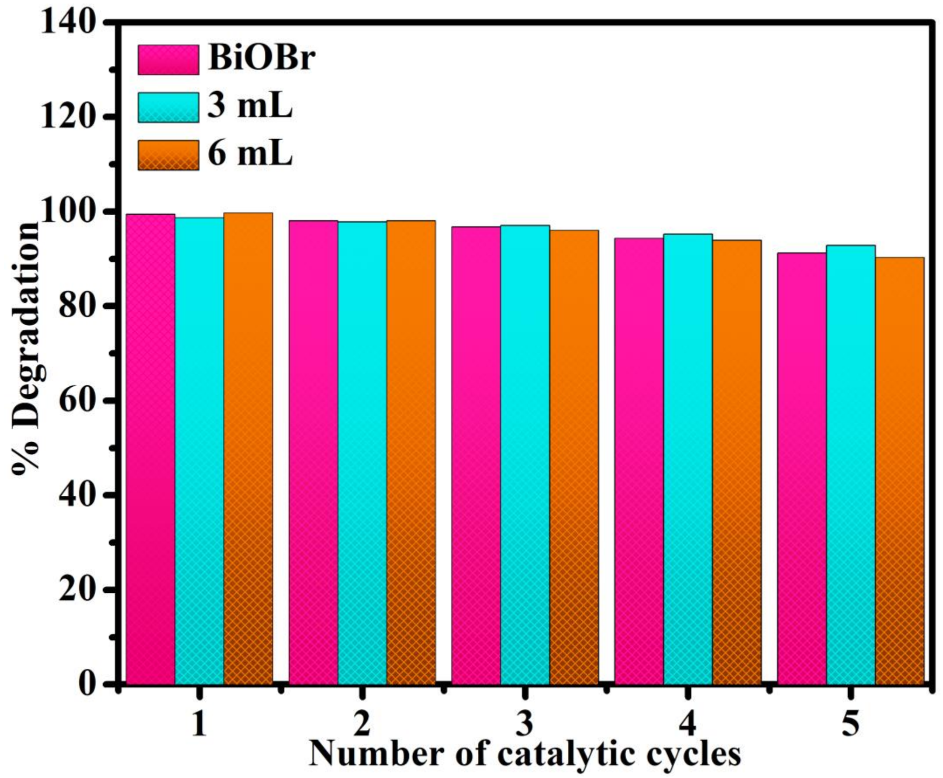

Fig.S5: Repeatability for five rounds of RhB dye de-colorization via BiOBr and (3 and 6 mL) Sm-g-C_3N_4 doped-QDs

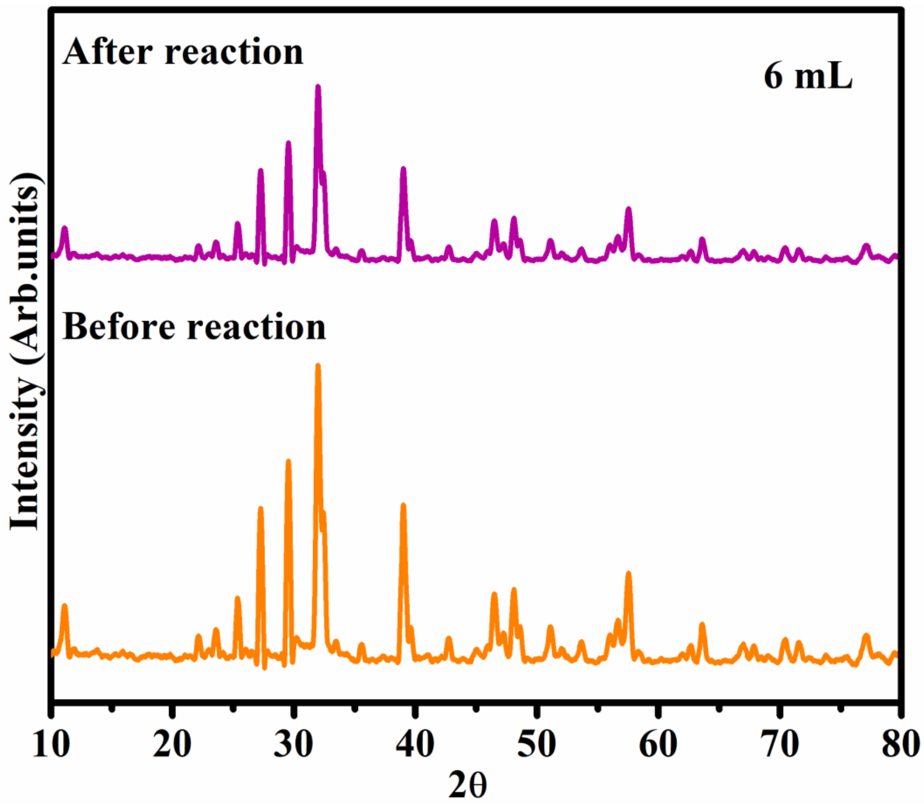

Fig.S6: Diffraction pattern of 6 mL Sm-g-C_3N_4 doped-QDs before and after reaction

Kinetic study

The degradation kinetics of the pollutant were studied using zero, first, and second-order reaction kinetics in this work. The individual equation was given as below (eq 1-3)

Zero-order kinetics

$$\frac{dc}{dt} = -k_0 \qquad (1)$$

First-order kinetics

$$\frac{dc}{dt} = -\,k_1C \qquad (2)$$

Second-order kinetics

$$\frac{dc}{dt} = -k_2C^2 \qquad (3)$$

Where k_0, k_1 and k_2 are the kinetic rate constants of zero, first and second order kinetics correspondingly; C is the concentration of RhB and t is the reaction time. The kinetic zero order model confirmed that the concentration of the RhB dye decreased as the time increased (Fig. S7a). The maximum degradation of RhB for BiOBr and (3 and 6 mL) Sm-g-C_3N_4 doped BiOBr occurred at 10 min. The correlation coefficient (R^2) values demonstrated very well fitting of the model with data (Table 1). The linear correlation graphing of $\ln(C_0/C_t)$ with time yields the fitting of the experimental data with the first-order model (Fig .S7b). In addition, the graphing of $(1/C_t)$ and time represents the second-order kinetics (Fig. S7c). Comparing the correlation coefficients (R^2) derived from Figs. S7(a-c), it can be shown that R^2 based on zero-order reaction kinetics (Fig. S7(a)) were 0.9989, 0.94947 and 0.99889 0.86785, which was much superior to R^2 based on first and second order. The findings showed that the degrading kinetics of RhB closely matched the zero-order kinetics.

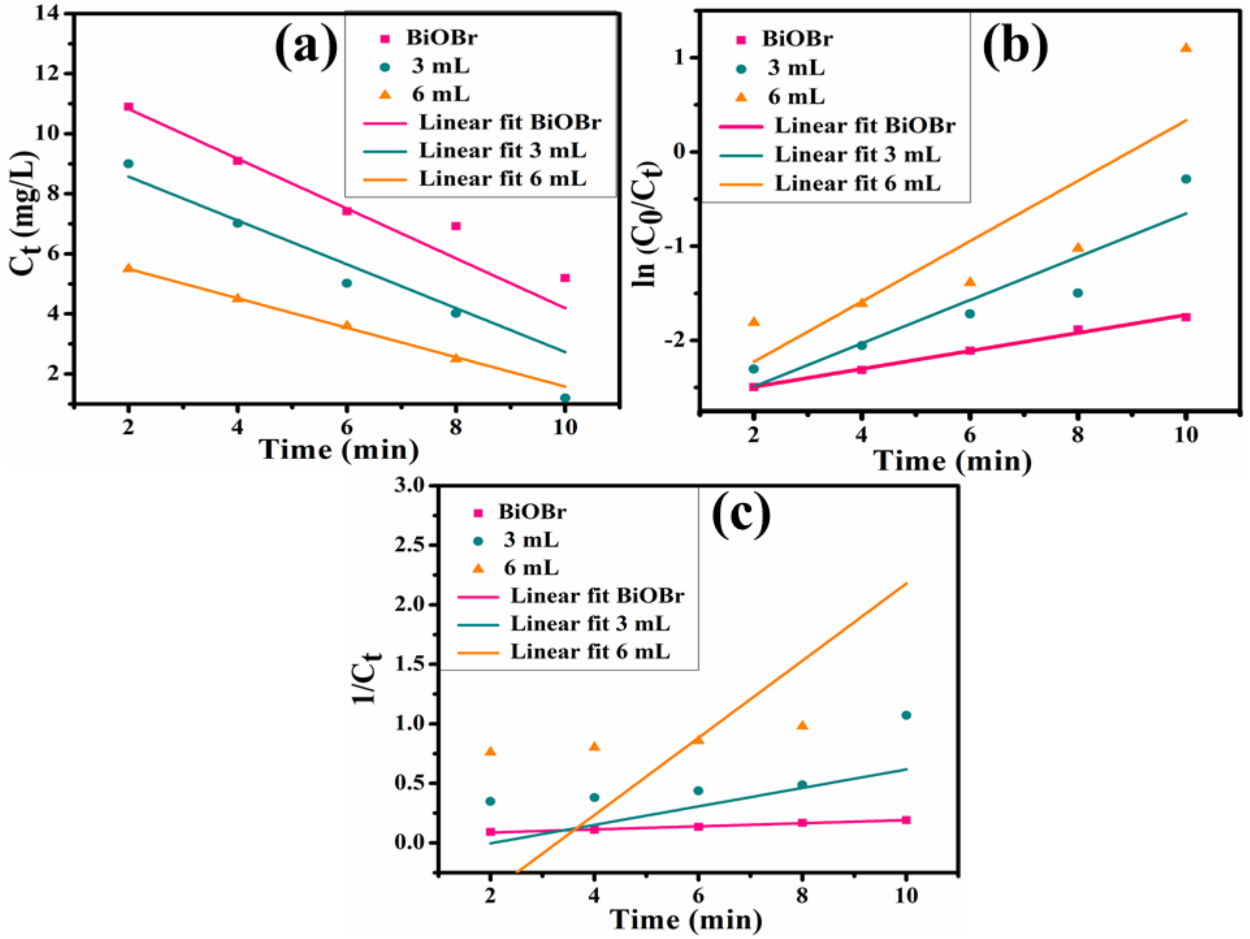

Fig. S7 (a) Zero-order kinetics, (b) first-order kinetics and (c) second-order kinetics

Table S1. Parameters of zero, 1st and 2nd order kinetic models.

**Kinetic model**	**Parameters**	**BiOBr**	**3mL Sm-g-C_3N_4/BiOBr**	**3mL Sm-g-C_3N_4/BiOBr**
Zero-order	K (mg/min)<br>R^2	0.829<br>0.99889	0.73<br>0.94947	0.49<br>0.99889
First-order	K_1 (min^{-1})<br>R^2	0.009551<br>0.9924	0.032026<br>0.64931	0.02296<br>0.81521
Second order	K_2 (L/mol min)<br>R^2	20.08081<br>0.98717	7.80384<br>0.5636	1.17578<br>0.41018

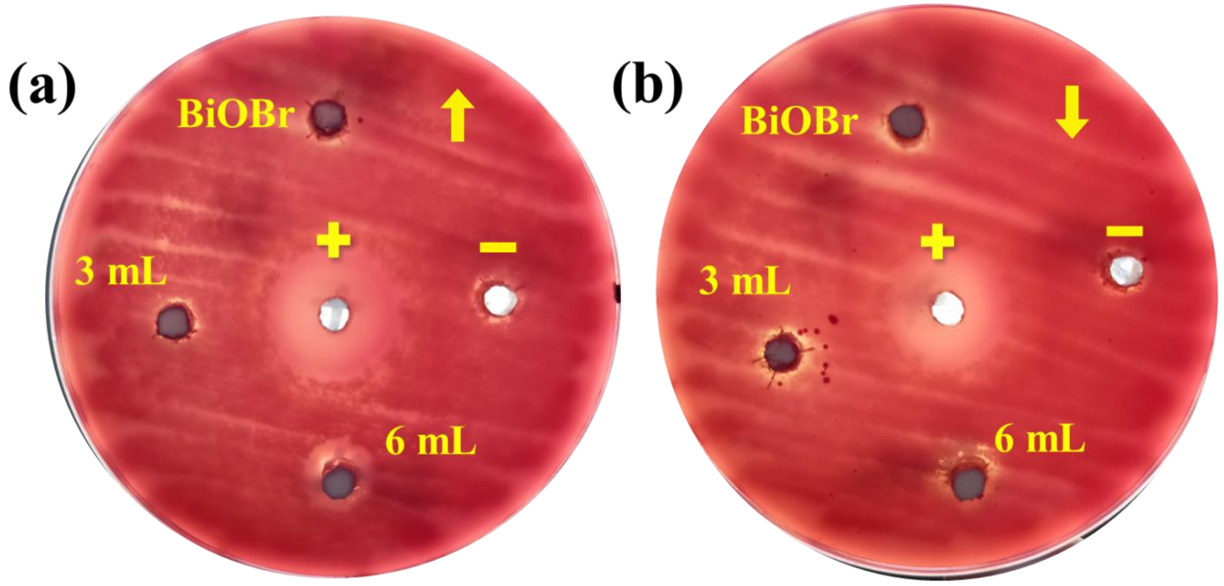

Fig.S8: In vitro evaluation for bactericidal action of prepared QDs contrary to (a) *E.coli* high (b) low concentration

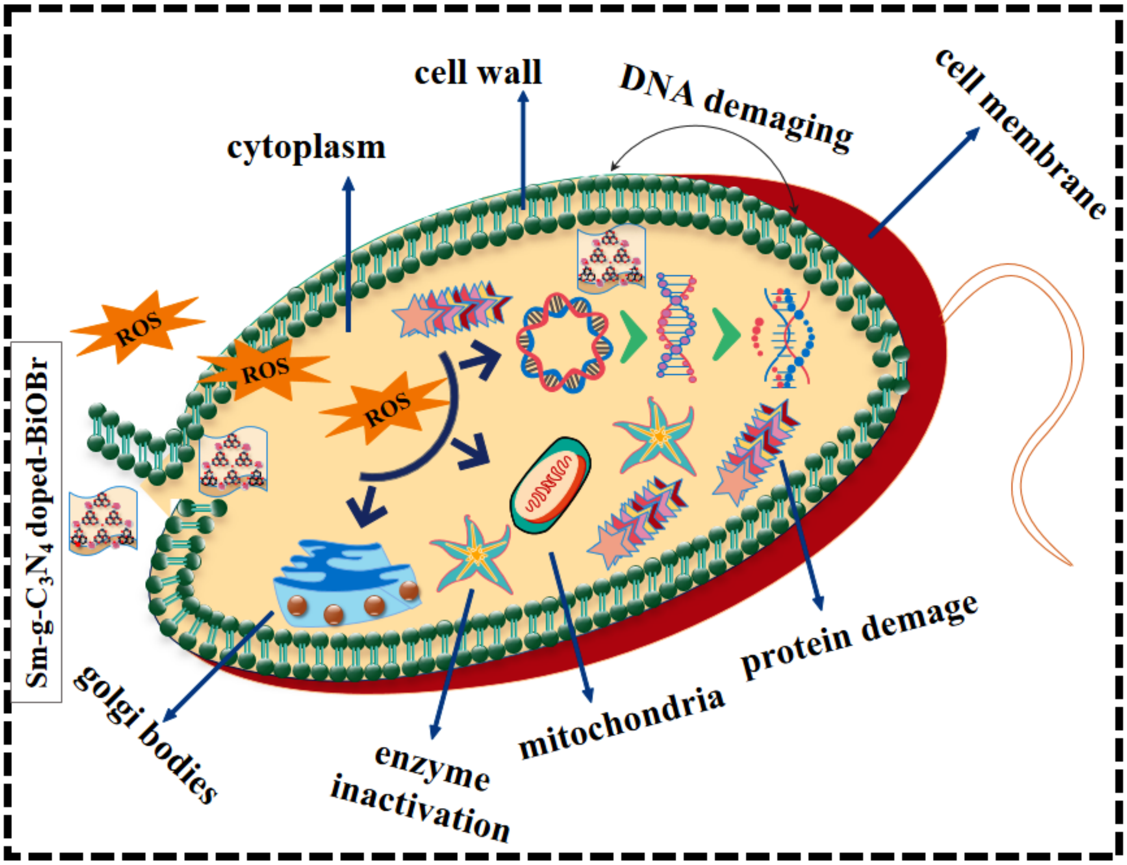

Fig.S9: Schematic of mechanism for antimicrobial action of synthesized Sm-g-C_3N_4 doped-BiOBr

Table.S2: describing a comparison of organic pollutants de-colorization rate with previous study

**Materials**	**synthesis routes**	**organic pollutants**	**degradation efficiency**	**references**
BiOBr/Ag nanocomposite	reduction	RhB, DCP,MO	60-80% after 240 min	[1]
flowerlike g-C_3N_4/BiOB	solvothermal	MO,RhB	98% within 90 min	[2]
hierarchical BiOBr microspheres	ionothermal	MB	55%	[3]
Nd/ BiOBr nanosheets	hydrothermal	POME	90.7 after four cycles	[4]
Sm-g-C_3N_4 doped-BiOBr	Co-precipitation	RhB	99.6% within 10 min	Present work

Table.S3: A comparison of microbicidal efficacy of current study with literature

**Bactericidal agents**	**synthesis routes**	**Bactericidal potential**	**references**
Pure BiOBr	Deposition precipitation	29.4% *E.coli*	[5]
3D-nano flower BiOBr	hydrothermal	44.23% *E.coli*	[6]
PET-Ti_3C_2/BiOBr/ppy textile	N/A	30.73% *S. aureus*	[7]
BiOBr microspheres	ionothermal	50% *M.lylea*	[3]
Sm-g-C_3N_4 doped-BiOBr	Co-precipitation	57% *E.coli*	Present work

REFERENCES

[1] M. Yaghoubi-berijani, B. Bahramian, S. Zargari, The Study of Photocatalytic Degradation Mechanism under Visible Light Irradiation on BiOBr/Ag Nanocomposite, Iran. J. Catal. 10 (2020) 307–317. http://ijc.iaush.ac.ir/article_677339_ebe3e1436ed82c4171bd1a2252b28b07.pdf (accessed May 16, 2023).

[2] M. Jiang, Y. Shi, J. Huang, L. Wang, H. She, J. Tong, B. Su, Q. Wang, Synthesis of Flowerlike g-C3N4/BiOBr with Enhanced Visible Light Photocatalytic Activity for Dye Degradation, Eur. J. Inorg. Chem. 2018 (2018) 1834–1841. https://doi.org/10.1002/ejic.201800110.

[3] D. Zhang, M. Wen, B. Jiang, G. Li, J.C. Yu, Ionothermal synthesis of hierarchical BiOBr microspheres for water treatment, J. Hazard. Mater. 211–212 (2012) 104–111. https://doi.org/10.1016/j.jhazmat.2011.10.064.

[4] J.C. Sin, C.A. Lim, S.M. Lam, H. Zeng, H. Lin, H. Li, A.R. Mohamed, Fabrication of novel visible light-driven Nd-doped BiOBr nanosheets with enhanced photocatalytic performance for palm oil mill effluent degradation and Escherichia coli inactivation, J. Phys. Chem. Solids. 140 (2020). https://doi.org/10.1016/j.jpcs.2020.109382.

[5] B. Liu, X. Han, Y. Wang, X. Fan, Z. Wang, J. Zhang, H. Shi, Synthesis of g-C3N4/BiOI/BiOBr heterostructures for efficient visible-light-induced photocatalytic and antibacterial activity, J. Mater. Sci. Mater. Electron. 29 (2018) 14300–14310. https://doi.org/10.1007/s10854-018-9564-4.

[6] Y. Zhao, Z. Li, J. Wei, X. Li, H. Shi, B. Cao, J. Fan, Efficient photodegradation of cefixime catalyzed by a direct Z-scheme CQDs-BiOBr/CN composite: Performance, toxicity evaluation and photocatalytic mechanism, Chemosphere. 292 (2022). https://doi.org/10.1016/j.chemosphere.2021.133430.

[7] J. Li, L. Ma, Z. Li, X. Liu, Y. Zheng, Y. Liang, C. Liang, Z. Cui, S. Zhu, S. Wu, Oxygen Vacancies-Rich Heterojunction of Ti3C2/BiOBr for Photo-Excited Antibacterial Textiles, Small. 18 (2022) 2104448. https://doi.org/10.1002/smll.202104448.
